# In *E. coli*, Interrupting One Death Pathway Leads You Down Another

**DOI:** 10.1371/journal.pbio.1001278

**Published:** 2012-03-06

**Authors:** Richard Robinson

**Affiliations:** Freelance Science Writer, Sherborn, Massachusetts, United States of America

## Abstract

A newly discovered apoptotic-like death is inhibited by the previously described mazEF-mediated death pathway, revealing two programmed cell death systems in *Escherichia coli.*

You might think that organisms would do well to just concentrate on staying alive, and leave death to the fates, but in fact, all eukaryotes have well-regulated programmed cell death pathways built into their genes, which help sculpt organs during development and scuttle infected cells before they get out of control. Bacteria, too, have cell death mechanisms, and, like so much of the bacterial world, they are more varied than their eukaryotic counterparts. In a new study in *PLoS Biology*, Hanna Engelberg-Kulka and colleagues describe a novel cell death pathway in *E. coli* that is inhibited by a second, and suggest that the two may serve different functions in the life, and death, of bacterial colonies.

One well-described bacterial death pathway involves the *mazEF* operon. The *mazF* gene encodes a stable toxin, which would kill the bacterium, except that it is cotranscribed with *mazE*, which encodes a labile antitoxin. As long as the cell is in good health, it stays that way. But if it becomes too damaged to produce more mazE antitoxin, the residual mazF will trigger cell death. But what happens when the *mazEF* system is disabled? This is the question the authors set out to investigate.

In the best-known type of eukaryotic programmed cell death, apoptosis, the cell membrane becomes depolarized, which can be detected by influx of a fluorescent dye. In contrast, *mazEF* death does not induce depolarization. But when the authors mutated *mazF* to block the pathway, and then subjected the bacteria to a lethal dose of DNA-damaging toxin, the bacterial cells still died, and in the process their membranes depolarized, and the cells became stained with dye. They termed this alternative death pathway apoptotic-like death (ALD). The same shunting to ALD occurred when *mazF* was left intact, but one or another of its downstream targets was deleted, indicating that disruption of any part of the *mazEF* system was sufficient to trigger this alternative pathway.

In bacteria, DNA damage triggers the so-called SOS response, which switches on a repair system. The response is mediated by the protein RecA. Since DNA damage also triggered ALD, the authors wondered whether RecA might also be at work in the ALD system. They showed that when activated by DNA damage, the *mazEF* system inhibited production of RecA, and prevented activation of the ALD system. In *mazEF* mutants, DNA damage (but not other stressors) led to a rise in RecA messenger RNA, triggering the ALD system, depolarizing the membrane, and killing the bacteria. But when they deleted *RecA*, DNA damage in *mazEF* mutants no longer induced membrane depolarization, reducing cell death.

What does it matter which way the bacterium dies? The authors suggest the answer lies in one final observation, that a quorum-sensing signal peptide called extracellular death factor (EDF) was also needed for *mazEF*'s inhibition of ALD. Quorum sensing is used by a population of bacteria to coordinate behaviors, including reproduction and stress response. When EDF was absent, DNA damage led to death by the ALD pathway, not the *mazEF* pathway.

The functional difference between the two pathways, the authors speculate, has to do with the survivors. A small percent of each colony lives through the crisis triggered by the DNA-damaging toxins. They suggest that, through the mechanism of quorum sensing, the *mazEF* system is cell-density dependent, and ensures that, in a crisis, enough bacteria die off to ensure a plenitude of bacterial raw materials in the environment for those that survive. The ALD pathway, in contrast, has no density dependence, and may serve as a backup system in the event the *mazEF* system fails.

It is also possible, they suggest, that the ALD system ensures that bacteria that have lost their “good-for-the-community” *mazEF* death response will nonetheless die off, preventing “cheaters” from exploiting the resources of their neighbors. The genetic basis of altruism is a controversial topic in evolution, and these results may provide new insights into how such a system can evolve.

**Erental A, Sharon I, Engelberg-Kulka H (2012) Two Programmed Cell Death Systems in** ***Escherichia coli*** **: An Apoptotic-Like Death Is Inhibited by the** ***mazEF*** **-Mediated Death Pathway. doi:10.1371/journal.pbio.1001281**

